# Numerical Modeling of Earthquake-Damaged Circular Bridge Columns Repaired Using Combination of Near-Surface-Mounted BFRP Bars with External BFRP Sheets Jacketing

**DOI:** 10.3390/ma12020258

**Published:** 2019-01-14

**Authors:** Xing-Gui Zeng, Shao-Fei Jiang, Xin-Cheng Xu, Hai-Sheng Huang

**Affiliations:** 1 Institute of Civil Engineering, Putian University, Putian 351100, China; yllzxg2012@163.com (X.-G.Z.); deepseawdd@163.com (H.-S.H.); 2 School of management, Fujian University of Technology, Fuzhou 350108, China; 3 College of Civil Engineering, Fuzhou University, Fuzhou 350108, China; 4 Jiangxi Province Architectural Design & Research Institute, Nanchang 330046, China; speedb13715@163.com

**Keywords:** reinforced concrete, bridge column, repair, numerical modeling, NSM BFRP, material damage-accumulation model

## Abstract

This paper reports the numerical simulation of earthquake-damaged circular columns repaired with the combination of near-surface-mounted (NSM) basalt fiber reinforced polymer (BFRP) bars with external BFRP sheets jacketing at quasi-static loading. The numerical modeling was carried out with the nonlinear OpenSees software platform by using the BeamWithHinges element. In the simulations, the effect of the previous earthquake damage on the behavior of the repaired columns was taken into account, and a simple and effective material damage-accumulation model is proposed to modify the constitutive of materials in the unrepaired regions of the repaired columns. The developed numerical models were validated by comparing their quasi-static findings with those obtained from a previous experimental program, and a good agreement can be observed. Furthermore, the efficiency of the repair technique used in tests is evaluated via the developed numerical model.

## 1. Introduction

Recent events [1,2] such as the Northridge earthquake in 1994, Kobe earthquake in 1995, Chi–Chi earthquake in 1999, and the Wenchuan earthquake in 2008, have repeatedly demonstrated the vulnerabilities of reinforced concrete (RC) bridge columns to seismic actions. Apart from the victims and the direct economic costs associated with the replacement of these damaged bridge columns, the disruption of crucial roads over a large time period created tremendous difficulties in the logistics of getting assistance to the impacted areas, therefore aggravating the initial consequences of the earthquakes. In the 2008 Wenchuan earthquake, some bridge columns suffered from severe damage and required either repair or demolition for reconstruction. If damaged bridge columns could be repaired and rehabilitated rapidly, it would be both more economical than demolishing and reconstructing the bridges and would also be extremely important for rescue efforts after an earthquake.

The use of fiber-reinforced polymers (FRPs) for repairing purposes of different types of concrete and RC elements [3,4,5,6,7], like beams and columns, is increasing nowadays and has been extensively investigated during the last years. However, in general, conducting experimental tests has many drawbacks. These include cost, time, difficulties and limitations in testing full scale members, and difficulties in implementing a parametric study on different variables. These shortcomings highlight the importance of developing finite element models which can offer effective ways to investigate the behavior of the repaired columns considering a wide variety of parameters. Thus, this paper develops a finite element model for the repaired columns based on the limited amount of experimental specimens.

One of the most crucial steps in establishing the finite element model is the adoption of appropriate material models for both the concrete and steel bars. Generally, modeling approaches can be classified into two categories [8]: the continuum method and the frame modeling method. According to the first one, the concrete area is modeled using solid elements while the steel bars are modeled using truss or beam elements. This approach leads to more accurate results but is time-consuming since the computational and modeling effort is large enough, especially for large scale RC structures. With regards to the frame modeling method, two different formulations have been proposed: the lumped plastic hinge method and the fiber model approach. These approaches appear to be more attractive in comparison with the continuum method, mainly due to their smaller computational and modeling efforts. For these reasons, these methods have been frequently adopted to examine the behavior of RC structures under seismic loadings [9,10]. The fiber model appears to be more refined and accurate in comparison with the lumped plastic hinge model, and thus it has been adopted in this paper. The fiber model involves the subdivision of any shape of concrete and steel bars’ composite section into small elements which are assumed to be in a state of uniaxial stress. Therefore, the fiber model needs only two uniaxial constitutive models, one for concrete and the other for steel bars.

For the numerical analysis of repaired columns, the key point is to simulate the original and unrepaired damage in the repaired columns. However, the damage for individual elements is calculated based on element data, such as element deformations, forces, or dissipated energy. These engineering parameters must be extracted from the solution and processed for calculating a damage index. A damage model is defined as an operator that calculates the damage index by applying a specific damage rule (e.g., Park–Ang damage model [11]). The damage index can be recorded for subsequent loss assessments, and in some cases, it may be used by the analysis components for degrading constitutive stiffness or strength parameters.

Selecting and calibrating an appropriate damage model for each RC component is a delicate task which requires knowledge of the available damage models as well as the monotonic and cyclic behavior of RC components. Damage in RC components may be caused by excessive deformation or by repeated load reversals. The damage in a RC component is associated with damage to the concrete (aggregate–cement matrix), reinforcement steel (rebar yielding, fracture, or buckling), bond loss, or a combination of all these elements. The damage models are categorized as non-cumulative and cumulative damage models based on the loading types and the failure mechanisms [12]. Non-cumulative damage indices use the envelope of maximum response, such as component ductility or loading, as the basic variable to calculate the damage. Non-cumulative models are not load path dependent, and they do not generally reflect the damage due to cyclic loading.

Cumulative damage models are employed to represent the damage under cyclic loading. Accumulated plastic deformation or the hysteretic energy is commonly used for calculating cumulative damage indices. The deformation-based cumulative damage models are mostly developed based on the low-cycle fatigue formulation, where large ductility excursions are sustained over many loading cycles [13,14,15,16,17]. Cumulative damage models based solely on energy was introduced by Kratzig et al. [18]. Inspired by Kratzig et al., Mehanny and Deierlein [19] proposed a damage index based on the accumulation of plastic deformations. On the basis of the Mehanny and Deierlein damage model, Gu et al. [20] defined the cumulative damage index for the materials of RC columns under cycle loading. In the Gu damage model [20], the ultimate displacement *d*_u_ under monotonic loading cannot be calculated by the existing equations but can be computed by finite element static push over analysis. This makes the damage index difficult to be employed in the practical engineering. Furthermore, the ultimate displacement *d*_u_ is obtained under monotonic loading rather than cyclic loading. However, in the seismic performance analysis, the bridge columns are under the cyclic loading, and it is reasonable to select the ultimate displacement under cyclic loading rather than monotonic loading to calculate the damage index. Thus, this paper modified the Gu damage index by replacing the ultimate displacement *d*_u_ under monotonic loading with the ultimate displacement under *d*^’^_u_ cyclic loading.

This paper proposes a modified Gu damage-accumulation model of materials in which the ultimate displacement *d*^’^_u_ under cyclic loading is calculated according to Eurocode 8 [21]. With the use of the proposed damage model to modify the constitutive model of concrete and steel in structures, the residual strength and stiffness of the materials in the structures can be calculated by considering the degradation of strength and stiffness after the structures suffer from cyclic loading. Afterwards, based on the fiber BeamWithHinges element in the OpenSees platform and the proposed damage model, a numerical model is generated for analyzing the seismic performance of repaired RC bridge columns with original and unrepaired damage. The proposed damage-accumulation model and the numerical model are verified by comparing the numerical simulation with the experimental results. Furthermore, the efficiency of the repair technique used in the tests is evaluated by the proposed numerical model. More specifically, the authors discuss the contribution of near-surface-mounted (NSM) basalt fiber reinforced polymer (BFRP) bars and longitudinal fibers of BFRP sheets to restoring the flexural strength of repaired columns and then presents the corresponding quantization design method of NSM reinforcements.

The rest of the paper is organized as follows. The OpenSees platform and models are reviewed and introduced in Section 2. Then, the numerical models of earthquake-damaged columns repaired with composite repair techniques are established by combining the proposed damage index into the existing material models to consider the stiffness and strength degradation in Section 3. In addition, the proposed numerical model and the corresponding damage index are verified by comparing the numerical analyses with the experimental results in Section 3. Some conclusive remarks are summarized in Section 4.

## 2. Overview on OpenSees Platform and Models

All analytical models performed herein were done using the OpenSees (Open System for Earthquake Engineering Simulation) platform [22,23]. The OpenSees platform is an open source software framework developed to simulate the response of structural and geotechnical systems subjected to earthquakes. For structural members, OpenSees performs fiber-based analysis. The flexural member is represented by unidirectional steel and concrete fibers which are assumed to be characterized by the selected material stress–strain relationship. The member stiffness and forces are obtained by numerically integrating the stiffness and forces of sections along the member length. The section deformation is used to obtain the strain in each fiber, based on the plane section assumption. The fiber stress and stiffness are updated according to the corresponding material models, followed by upgrading of the section force resultant and the corresponding stiffness [24].

### 2.1. Structural Elements’ Modeling

**Figure 1:**
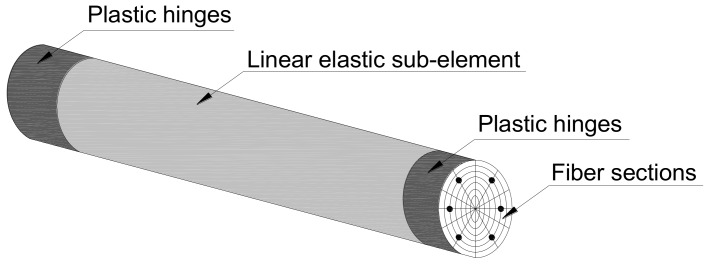
The sketch of the BeamWithHinges element.

OpenSees has several models for describing the cyclic behavior of RC structural elements, such as the BeamWithHinges element and the Nonlinear Beam-Column element, and these elements have been used for simulating the RC member by numerous scholars [25,26,27]. The BeamWithHinges element, available in OpenSees, was used to simulate the response of the RC column under study. Plasticity is concentrated over specified hinge lengths at the element ends and each BeamWithHinges element is divided into three parts: two hinges at the ends and a linear-elastic region in the middle (see Figure 1). By contrast with other types of distributed plasticity elements in which the Gauss integration points are distributed along the element length, the BeamWithHinges element localizes the integration points in the hinges’ regions.

### 2.2. Materials Models

**Figure 2:**
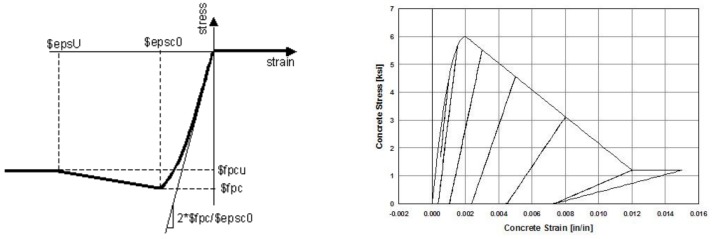
Material parameters for the *Concrete*01 model [23].

**Figure 3:**
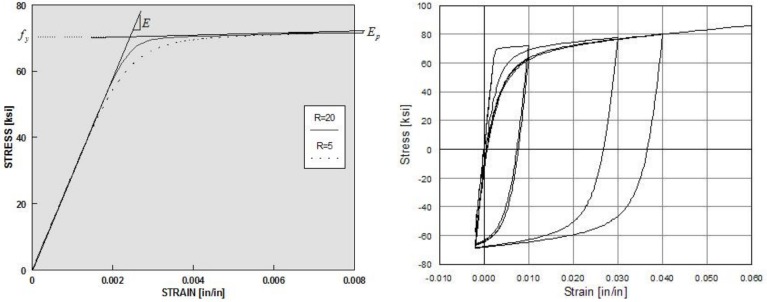
Hysteretic response of steel bars with the *Steel*02 model [30].

**Figure 4:**
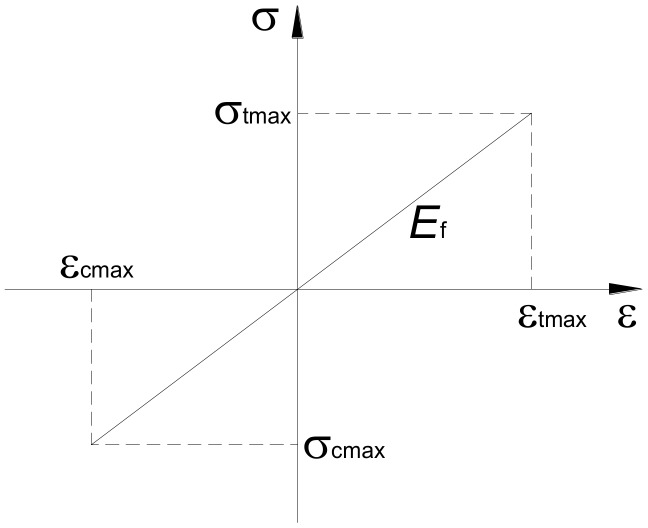
Material parameters for *Elastic Material* model.

OpenSees has several available material models for describing the monotonic and cyclic response based on hysteretic rules of concrete and steel reinforcement fibers. In the numerical modeling of the RC column presented in this work, the following material models were used: *Concrete*01 for the concrete (Figure 2) and *Steel*02 for the steel (Figure 3). The *Concrete*01 model (Figure 2) was adopted for the concrete fibers in the BeamWithHinges element. The concrete models take into account the confinement effect due to the stirrups and are based on the law proposed by Hognestad [28] adapted by Guedes [29]. The *Concrete*01 model does not take into account the concrete tensile strength. For steel bars in the column modeling, the *Steel*02 material model was assigned to the BeamWithHinges element. The *Steel*02 model is based on the Giuffré–Pinto formulation, implemented later by Menegotto and Pinto [30]. With regards to the FRP material, its characteristic is linear elastic. Therefore, it is reasonable to adopt the *Elastic Material* model as shown in Figure 4. Considering the ultimate strain for FRP materials, this paper combines the *Elastic Material* model with the *MinMax Material* model to define the minimum and maximum ultimate strain for FRP material. 

## 3. Numerical Modeling of Earthquake-Damaged Columns Repaired with Composite Repair Technique

### 3.1. Brief Description of the Tested Column and Cyclic Test Procedure

The main objective of the experimental program was the evaluation of the NSM BFRP bars as a flexural repairing system for earthquake-damaged RC circular bridge columns [31]. To study the seismic performance of earthquake-damaged bridge columns repaired by local NSM BFRP bars and external BFRP sheets jacketing, the reserved four earthquake-damaged circular bridge columns were repaired by local NSM BFRP bars and external BFRP sheets jacketing.

#### 3.1.1. Review of Original Columns

**Figure 5:**
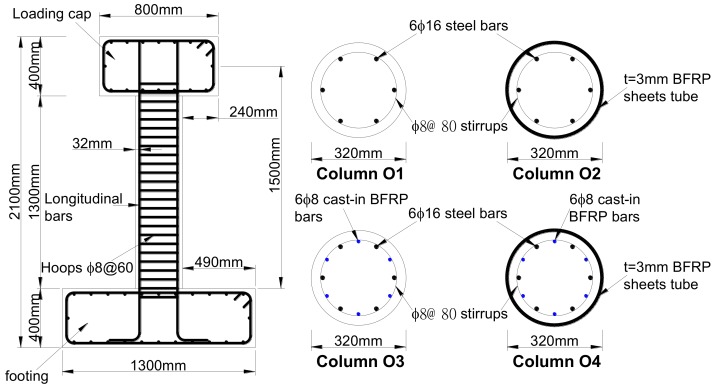
Geometry and reinforcement details of original columns.

**Figure 6:**
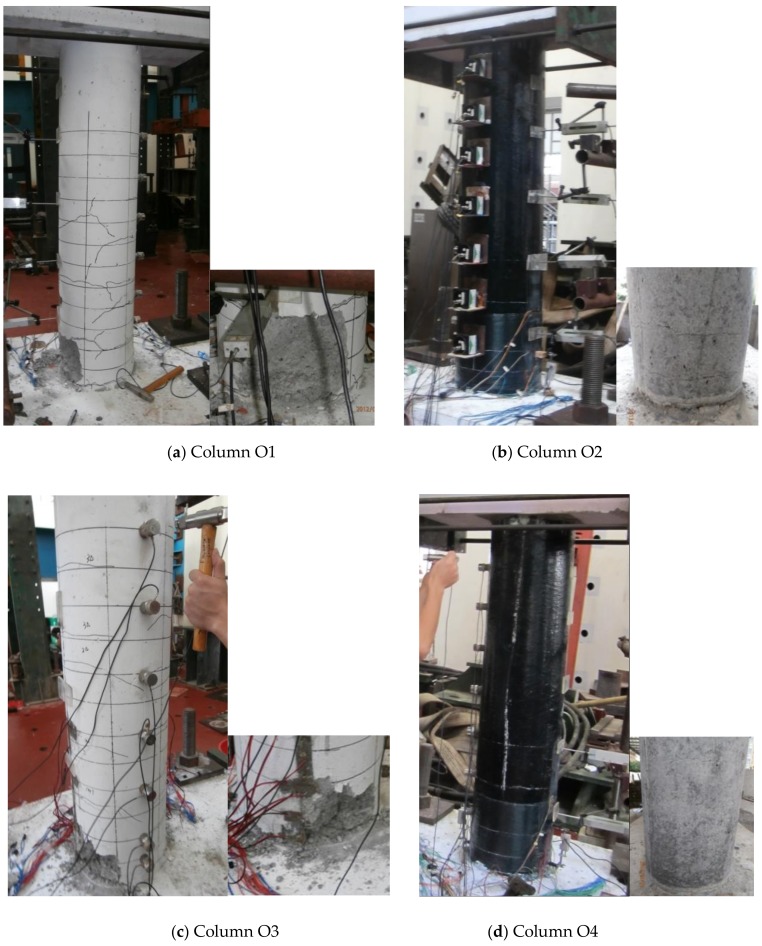
Ultimate failure modes of the original columns.

The geometry and reinforcement details of the original columns [32] before damage are shown in Figure 5. The average compressive strength of the concrete measured at the original test date was 20.85 MPa (cylindrical compressive strength of 16.68 MPa). The mechanical properties of the other materials provided by the manufacturer are shown in Table 1. The four original bridge columns were tested to failure under cyclic lateral loading and a constant axial load of approximately 161 kN to simulate the vertical load from the superstructure, and all tests were conducted at the Structural Laboratory of Civil Engineering College of Fuzhou University in Fuzhou, China. Figure 6 shows the damaged columns after the original tests. Based on the visual observations and measured response data after the completion of the test, the damage to all four original columns included concrete cracking, cover spalling, and all longitudinal reinforcement yielding. In addition, the damage to all four original columns was concentrated near the base of the column at the location of maximum moment due to the flexure-dominant behavior in columns.

#### 3.1.2. Review of the Repair Design and Cyclic Test Procedure

To repair the damaged columns mentioned in Section 3.1.1, which were tested to failure under cyclic lateral loading, the four damaged columns were repaired by using the NSM BFRP bars and external BFRP sheets jacketing. Moreover, to maximize the time efficiency, only the region of the columns at the plastic hinge region where the damage was concentrated was repaired. Portions of the columns outside this region exhibited slight cracks on the concrete surface but were not repaired and were without treatment of the core concrete and the damaged steel bars in the plastic hinge region. The parameters of the repair design were considered by varying the layer number of BFRP sheets for three or five layers and the diameter of BFRP bars from 8 mm to 10 mm. The detailed information of the repair design is presented in Table 2.

**Figure 7:**
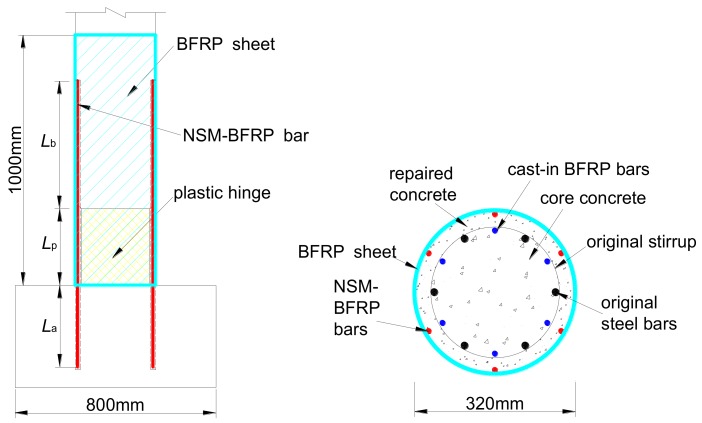
Repair techniques for the damaged specimens.

The detailed design information of the NSM BFRP bars is given in Figure 7. This paper adopts the anchorage length *L*_a_ equal to 40 times the diameter of the BFRP bar. The bonding length *L*_b_ beyond the plastic hinge length is equal to 50 times the diameter of the BFRP bars (50*d*_f_).

**Figure 8:**
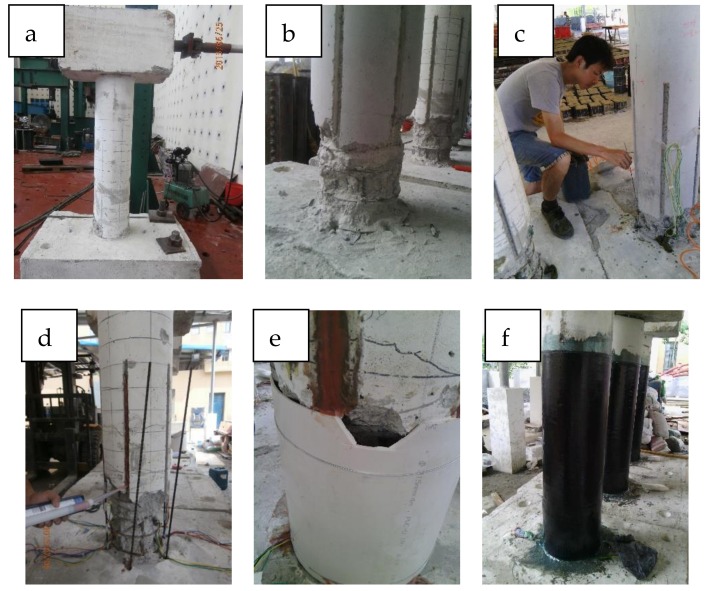
Repair procedure: (**a**) the column after straightening; (**b**) the column after the loose concrete removal and chiseling grooves; (**c**) clearing the holes and grooves; (**d**) injecting epoxy adhesive and placing the NSM BFRP bars; (**e**) the repair mortar placement; and (**f**) the BFPR sheet application.

For the four repaired columns, the BFRP sheets were 1 m wide and with a nominal thickness of 0.18 mm per ply. The properties of the BFRP bars and the BFRP sheets provided by the manufacturer are given in Table 1, and it is noted that the stress–strain relationship of the fibers is linear-elastic until failure. With regards to the repair mortar, the average compressive strength of the repair concrete used to replace the removed damaged concrete at the test data based on the results of the three tests measured in accordance with GB/T 50081-2002 [33] was 28.82 MPa. The repair procedure consisted of six steps (including instrumentation application) shown in Figure 8.

**Figure 9:**
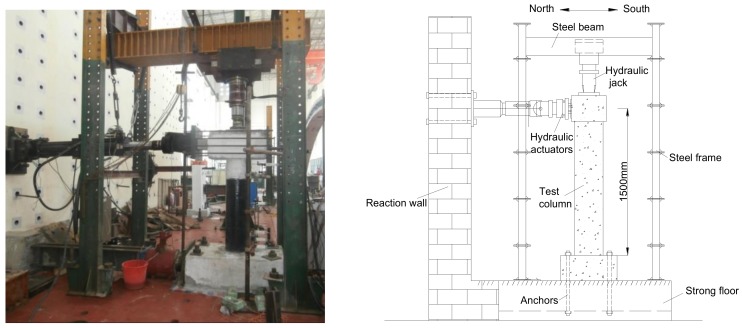
Test setup.

The repaired columns were tested under the combined effects of a constant axial compression and incrementally increasing lateral deformation reversals. Figure 9 illustrates the test setup. As with the original column, an axial load of 161 kN was used, corresponding to an axial load ratio of 12%. Following the application of the axial compression, the columns were then subjected to lateral cyclic loading that consisted of successive cycles incrementally increasing by 8 mm for columns R1 and R3 and by 10 mm for columns R2 and R4 of displacement amplitudes in each direction: Three cycles were applied at each load stage, and the load rate was 1 mm/s. Furthermore, for the repaired columns R1 and R3 and for columns R2 and R4, the lateral displacement was repeated for one cycle before the drift ratio reached 0.53% (8 mm) and 0.67% (10 mm) with the increment of 1 mm, respectively. After that, three cycles of the lateral displacement were applied.

#### 3.1.3. Test Results of Repaired Columns

**Figure 10:**
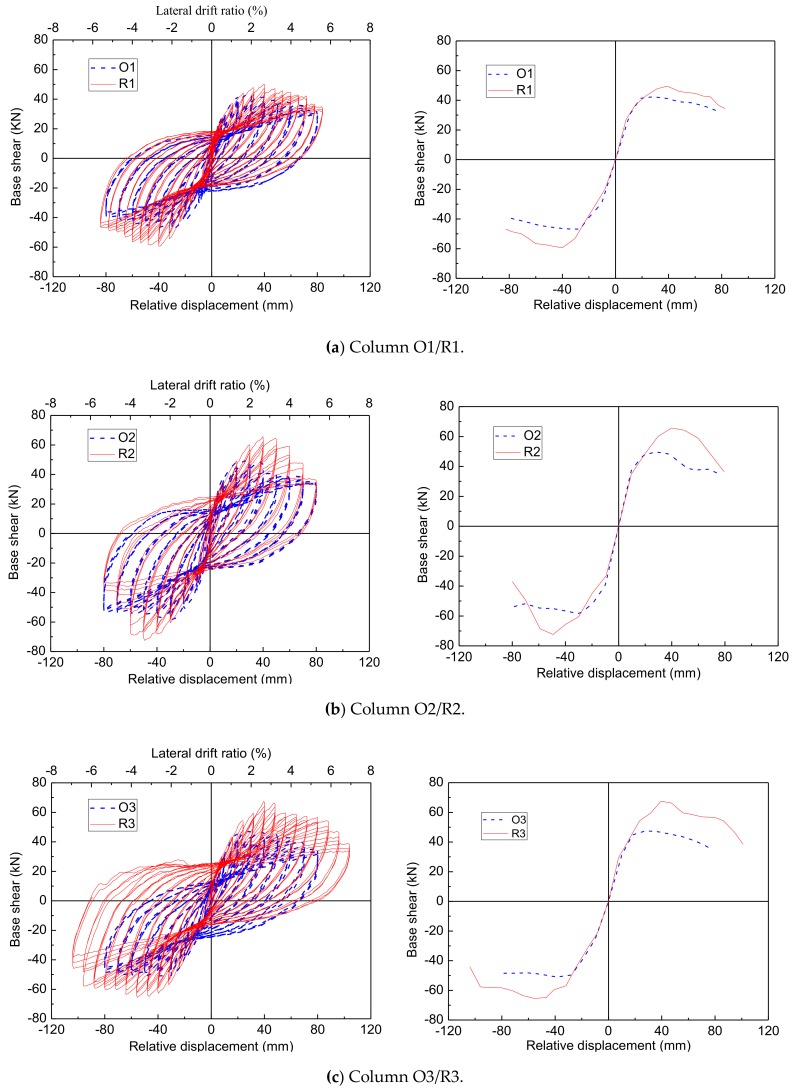

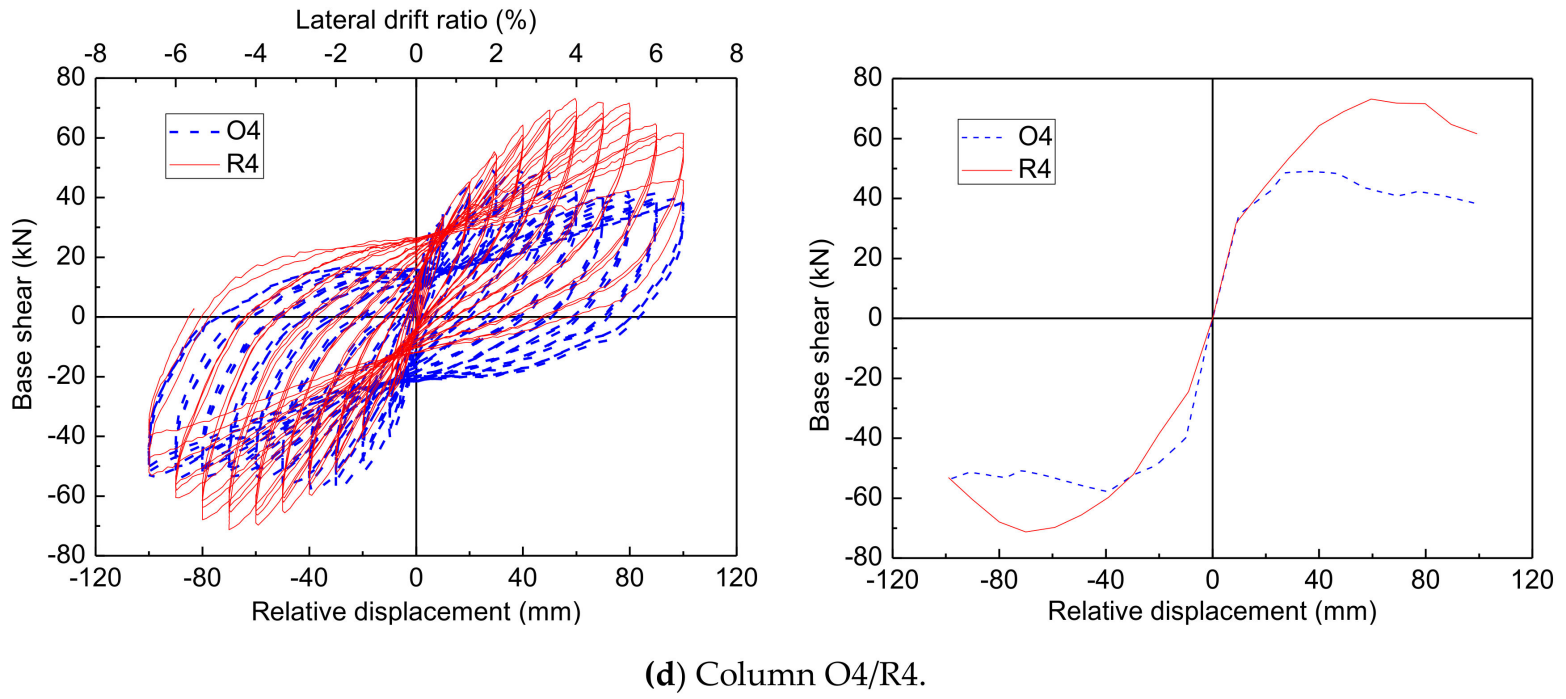
The hysteretic curves and the corresponding envelopes for columns: (**a**) Column O1/R1; (**b**) Column O2/R2; (**c**) Column O3/R3; (**d**) Column O4/R4.

The main results are shown in Figure 10, namely the hysteresis load–displacement relationship and the corresponding load–displacement envelopes. The strength, stiffness, and ductility of original and repaired columns are summarized in Table 3, and the comparison of results of the original columns with those of the repaired columns are summarized in Table 4. The strength index for all four columns varied between 120.49% and 133.47%. The strength was restored or even enhanced compared to that of the original columns. The stiffness index ranged from 86.86% to 110.14%. The stiffness of the repaired columns (columns R1 and R4) was not fully restored due to the stiffness degradation of the reinforcing bars and concrete cracking in the unrepaired portions of the columns. With regards to the ductility index, they ranged from 68.44% to 112.04%. This indicates that the ductility was unable to be restored fully. However, based on the latest Caltrans Seismic Design Criteria, SDC version 1.4 [34], a minimum displacement ductility capacity of 3 is required for ductile members. The more detailed discussion of the experiment results can be seen in reference [31]

The experimental results of the repaired columns test were used to calibrate the adopted numerical model, as described in Section 3.2. The numerical analysis results are described in Section 3.3.

### 3.2. Numerical Model

As discussed in the previous section, the column stiffness was not fully restored during the repair due to the existing damage in the repaired columns. Therefore, standard analysis methods could not be used directly for modeling the repaired columns, and a new numerical model had to be developed for the repaired columns.

#### 3.2.1. Damage Evaluation of Original Columns Damaged by Cyclic Loading

It can be seen from the repair design in Section 3.1 that portions of the columns outside the plastic hinge region were not repaired, and the core concrete and the damaged steel bars in the plastic hinge regions are without treatment. This implies that the original damage still exists in the repaired columns, even after repairing. This is why the stiffness of columns was not fully restored during the repair as shown in Section 3.1. Thus, the original and unrepaired damage should be considered when simulating the repaired columns.

For the numerical simulation of the repaired columns, the crucial issue is that of evaluating the damage condition of the original columns under cyclic loading. However, the damage for individual elements is calculated based on element data, such as element deformations, forces, or dissipated energy. These engineering parameters must be extracted from the solution and processed for calculating a damage index. A damage model is defined as an operator that calculates the damage index by applying a specific damage rule. The damage index can be recorded for subsequent loss assessment, and in some cases, it may be used by the analysis components for degrading constitutive stiffness or strength parameters.

Most damage indices are cumulative in nature, reflecting the dependence of damage on both the amplitude and the number of cycles of loading. It is well-known that the stiffness and strength of the materials in RC structures will degrade seriously when cyclic loading ranges between the tensile and the compressive. On the basis of the Mehanny damage model [19], Gu et al. [20] proposed an accumulative damage index for the materials of RC members with the flexural failure, and then the damage index is integrated into restoring force model of steel bars and concrete, respectively.

(1) $$D=\frac{{\left(d_{\max}\right)}^{\alpha }+{\left(\sum d_{i}\right)}^{\beta }}{{\left(d_{u}\right)}^{\alpha }+{\left(\sum d_{i}\right)}^{\beta }}$$

where *d*_i_ is the absolute value of displacement for each cycle i, *d*_max_ is the maximum displacement in all cycles, and *d*_u_ is the ultimate displacement under monotonic loading. The *α* and *β* are the constant parameters, which are equal to 3.0 and 0.2 for concrete materials and 0.5 and 1.0 for steel material, respectively, as described in the reference [19].

From the Equation (1), it is found that the ultimate displacement *d*_u_ needs to be determined in advance because it needs to be calculated by finite element static push over analysis since the ultimate displacement *d*_u_ under monotonic loading cannot be calculated by existing equations. This will enhance the inconvenience for the application of the Gu damage index. In addition, the ultimate displacement *d*_u_ is obtained under monotonic loading. However, this paper focuses on the seismic performance of the column under cyclic loading. Thus, it is necessary to replace the ultimate displacement *d*_u_ under monotonic loading with the one *d*^’^_u_ under cyclic loading to modify the Gu damage index. For the *d*^’^_u_ under cyclic loading, Fardis and coworkers [35,36] assembled a comprehensive database of experimental results of RC element tests and developed empirical formulas for yield and ultimate drifts. A version of their expressions has been adopted in Eurocode 8, Part 3 [21]. As shown in Eurocode 8, the value of the ultimate chord rotation *θ*_u_ at the ultimate displacement *d*^’^_u_ of RC members under cyclic loading may be calculated from the following expression:

(2) $$\theta_{u}=\frac{1}{\gamma_{el}}0.016\times \left(0.3^{v}\right){\left[\frac{\max\left(0.01,\omega^{\prime }\right)}{\max\left(0.01,\omega \right)}f_{c}^{'}\right]}^{0.225}{\left(\frac{L_{V}}{h}\right)}^{0.35}25^{c}\times 1.25^{100\rho_{d}}$$

where *γ*_el_ equals 1.5 for primary elements and 1.0 for secondary elements; *h* is the depth of cross section (equal to the diameter *D* for circular sections); *v* = *N*/*bhf*_c_ (*b* width of compression zone and *N* axial force positive for compression); *ω* and *ω*^’^ are the mechanical reinforcement ratios of the tension (*ω* = *ρf*_y_/*f*_c_) and compression (*ω*^’^ = *ρ’f*_y_/*f*_c_), respectively, longitudinal reinforcements, where *ρ* and *ρ’* are the ratios of the tension (including web reinforcement) and compression reinforcements normalized to bd and *f*_c_ is the uniaxial (cylindrical) concrete compressive strength (MPa); *L*V/*h* is the shear span ratio of the section of maximum moment; *c* = *αρ*_sh_*f*_yw_/*f*_c_, where *ρ*_sh_ = *A*_sh_*/b*_w_*s*_h_ is the ratio of transverse steel parallel to the direction of loading (*s*_h_ = stirrup spacing) and *f*_yw_ is the yield strength of the transverse steel; and *ρ*_d_ is the steel ratio of the diagonal reinforcement (if any), in each diagonal direction. *α* is the confinement effectiveness factor that may be taken equal to the following equation:

(3) $$\alpha =\left(1-\frac{s_{h}}{2b_{c}}\right)\left(1-\frac{s_{h}}{2h_{c}}\right)\left(1-\frac{\sum b_{i}^{2}}{6h_{c}b_{c}}\right)$$

where *b*_c_ and *h*_c_ are dimensions of the confined core to the inside of the hoop and *b*_i_ is the centerline spacing of the longitudinal bars (indexed by i) laterally restrained by a stirrup corner or a crosstie along the perimeter of the cross section.

It can be seen in Section 3.1 that the columns O3 and O4 were designed as hybrid reinforced concrete columns. Thus, it is logical to consider the effect of BFRP bars on the *ω* and *ω*^’^. However, in Eurocode 8, the *ω* and *ω*^’^ just take into account the steel bars without regard to the FRP bars. Thus, the *ω* and *ω*^’^ should be changed to suit the hybrid reinforced concrete columns with BFRP bars, as follows:

First, as the same steel bars, the ration of NSM reinforcement *ρ*_f_ can be divided into two parts of the tension *ρ*_ft_ and compression *ρ*_fc_. Then, the ratio of BFRP bars should be substituted for the ratio of steel bars by using the equal strength method *ρ*_f_^’^ = *ρ*_f_*f*_f_/*f*_y_, and the corresponding ratios of the tension *ρ*_ft_^’^ and compression *ρ*_fc_^’^ can be obtained. Finally, the expression of *ω* and *ω*^’^ is given by the sum of two terms—one to account for the contribution of steel bars and a second one to account for the contribution of BFRP bars—as follows:

(4) $$\{\begin{array}{c} \omega =\rho \frac{f_{y}}{f_{c}}+\rho_{\mathrm{ft}}^{'}\frac{f_{y}}{f_{c}}\\ \omega^{\prime }=\rho^{\prime }\frac{f_{y}}{f_{c}}+\rho_{\mathrm{fc}}^{'}\frac{f_{y}}{f_{c}} \end{array}$$

For the parameter *c* in Equation (4), it is mainly considering the confinement effects of the stirrups on the concrete. However, as mentioned in Section 3.1, the columns O2 and O4 were design as BFRP tubular columns, and it is logical to adopt the expression in Equation (4), with *c* given by the sum of two terms—one to account for the contribution of stirrups and a second one to account for the contribution of the jacket—as follows (Bournas et al. [37]):

(5) $$c=\alpha \rho_{\mathrm{sh}}\frac{f_{\mathrm{yw}}}{f_{c}}+\alpha_{f}\rho_{\mathrm{fh}}\frac{f_{\mathrm{fe}}}{f_{c}}$$

where *ρ*_fh_ = 2*n*_f_*t*_f_/*b*; *n*_f_ is the number of layers of the fiber sheet; *t*_f_ is the thickness of per fiber sheet; *f*_fe_ is the effective strength of jacket; and *α*_f_ is the effectiveness coefficient for confinement with fibers, equal to

(6) $$\alpha_{f}=\beta \left[1-\frac{{\left(b-2R\right)}^{2}+{\left(h-2R\right)}^{2}}{3bh}\right]$$

where *R* is the radius at the corners of the cross section for rectangular columns and is the radius of the cross section for circular columns. For the FRP material, the coefficient *β* is taken as equal to 1.

After determining the value of the ultimate chord rotation *θ*_u_, the ultimate drift *d*^’^_u_ can be calculated by Equation (7). Thus, the *θ*_u_ and *d*^’^_u_ for the four original columns after cyclic loading are summarized in Table 5.

(7) $$d_{u}=\theta_{u}L$$

in which the *L* is the effective height of the columns. 

Submitting Equation (7) into Equation (1) by replacing the *d*_u_ with *d*^’^_u_, the modified Gu damage index *D* with the same form Equation (1) can be derived.

Then the damage index for both the concrete (*D*^’^_c_) and steel bars (*D*^’^_s_) of the four original columns can be obtained. According to the modified damage index calculated, the residual strength and stiffness of the concrete and steel bars considering the degradation of the material property are calculated for the original column under the cyclic loading as follows (Gu et al. [20]):

(1) Concrete
  (8) $$\{\begin{array}{c} f_{c}^{\prime }=\left(1-\xi_{\mathrm{Fc}}D_{c}^{\prime }\right)f_{c}\\ E_{c}^{\prime }=\left(1-\xi_{\mathrm{Kc}}D_{c}^{\prime }\right)E_{c} \end{array}$$
(2) Steel bar
  (9) $$\{\begin{array}{c} f_{y}^{\prime }=\left(1-\xi_{\mathrm{Fs}}D_{s}^{\prime }\right)f_{y}\\ E_{s}^{\prime }=\left(1-\xi_{\mathrm{Ks}}D_{s}^{\prime }\right)E_{s} \end{array}$$

in which *f*_c_ and *f*_y_ are the original strength of the concrete and steel bars in the column, respectively, while *f*_c_’ and *f*_y_’ are the residual strength of the concrete and steel bars in the column after cyclic loading; *E*_c_ and *E*_s_ are the original stiffness of the concrete and steel bars in the column, respectively; and *E*_c_’ and *E*_s_’ are the residual stiffness of the concrete and steel bars in the column after cyclic loading. According to the Reference [20], *ξ*_Fc_ and *ξ*_Fs_ are equal to 0.27, while *ξ*_Kc_ and *ξ*_Ks_ are equal to 0.15.

Consequently, the mechanical properties for the concrete and steel bars considering the degradation of strength and stiffness are calculated in terms of the Equations (8) and (9) and are summarized in Table 6. To verify the accuracy and reliability of the modified damage index, these mechanical properties were adopted in the numerical models, which were calibrated by the experimental results in Section 3.3.

#### 3.2.2. Modeling Strategy

##### Structural Elements’ Modeling

**Figure 11:**
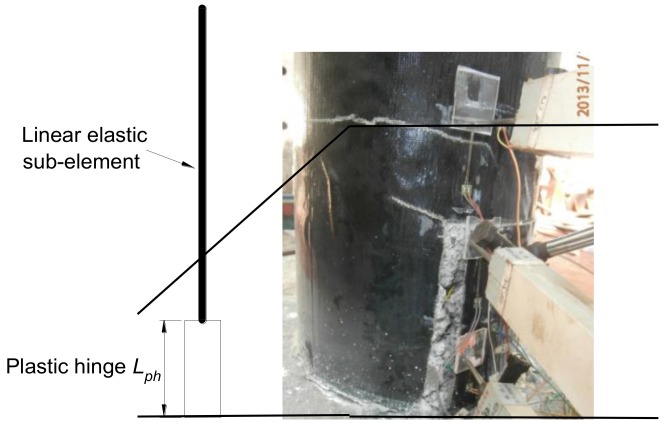
Plastic hinge’s location.

The model of the column was built with the BeamWithHinges elements. For the bridge column, the plastic hinge is easy to form at the column base. Figure 11 shows the location of the plastic hinge of the original columns formed during the cyclic loading. In terms of the visual observation, the damage in the original columns concentrates within the plastic hinge region, with slight cracks on the concrete surface outside the plastic hinge region.

The plastic hinge length *L*_ph_ [38] incorporates the strain penetration length, *L*_sp_, which is a function of the yield stress and diameter of the longitudinal reinforcement. The equations for the strain penetration length and plastic hinge length may be seen in Equations (10) and (11), respectively.

(10) $$L_{\mathrm{sp}}=0.022f_{y}d_{\mathrm{bl}}$$

(11) $$L_{ph}=kL_{c}+L_{\mathrm{sp}}$$

(12) $$k=0.2\left(\frac{f_{u}}{f_{y}}-1\right)\leq 0.08$$

where the *L*_c_ is the effective length of column, *f*_y_ is the yield stress of longitudinal reinforcement, *f*_u_ is the ultimate tensile stress of the longitudinal reinforcement, and *d*_bl_ is the diameter of longitudinal reinforcement. Following Equation (12), the height of the plastic hinge is 230 mm. While the actual plastic hinge length needs to consider the practical damage degree of columns, the adopted length in the numerical model for the plastic hinge region corresponds to the values measured in the test, i.e., 280 mm for the column O1, 300 mm for the column O2, 300 mm for the column O3, and 300 mm for the column O4.

**Figure 12:**
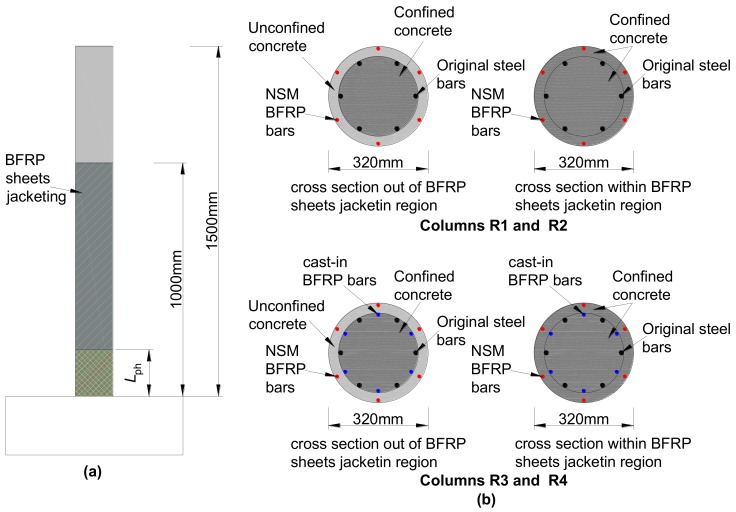
Model of repaired column: (**a**) column element dimensions and (**b**) fiber modeling at section level.

Figure 9 illustrates the RC column numerical model built-up and implemented within the OpenSees platform. Different colors can be distinguished from Figure 12, each one corresponding to a different material used in the computational model. The model of the column was built with the BeamWithHinges element. The fiber section used for the column element is discretized into core fibers (assumed to be confined by the stirrup and BFRP sheets jacketing) and cover fibers (unconfined).

The next section provides the description of the assumed values for the material properties assigned to the BeamWithHinges element in the numerical analyses.

##### Materials

(1) Concrete

In the OpenSees platform, the *Concrete*01 model was adopted for the concrete fibers in the the BeamWithHinges element. The concrete models take into account the confinement effect due to the stirrups. The *Concrete*01 model does not take into account the concrete tensile strength. The use of a concrete constitutive model with no tensile strength is assumed to be appropriate for the modeling of the repaired columns due to the fact that the existence of damage in the repaired columns results in the loss of the concrete tensile strength but not to cause any other significant nonlinear behavior in the column. Thus, it is reasonable to adopt the *Concrete*01model for concrete.

According to the repair design mentioned in Section 3.1, the core concrete in the plastic hinge was not repaired and was without any treatment to the steel bars. Thus, the degradation of the strength and stiffness should be considered for the concrete and steel bars by using the proposed damage index. However, the strength and stiffness of the concrete and the steel bars in the column outside of the plastic hinge region were not modified since the materials in this region were with slight damage. As discussed in the previous section, the cover concrete was replaced with the repair mortar in the plastic hinge region of the repaired columns. Therefore, the BFRP sheets jacketing confined properties were calculated for the core and cover concrete separately using the original concrete and repair mortar properties, respectively. Similarly, the core and cover concrete of the region outside the plastic hinge region but inside of the external BFRP sheets jacketing should take into account the confinement effect of the BFRP sheets jacketing by using the original concrete. Furthermore, the core concrete in both the plastic hinge and outside of the plastic hinge regions should take into account the confinement effect of the stirrup and BFRP sheets jacketing. Table 7 summarizes the parameters for the original and repaired concrete mechanical models adopted in the numerical analyses according the Kent and Park model [39,40]. In addition, for the core concrete in the plastic hinge region, the strength and stiffness should take into account the damage, and the corresponding values employed in the simulation of repaired columns are shown in Table 6.

(2) Steel

For steel bars in the column modeling, similar to the concrete, the strength and stiffness of the steel bars in the plastic hinge of the columns should be modified by using the proposed damage index but without any modification for the strength and stiffness of the steel bars outside of the plastic hinge. The mechanical parameters for the steel bars of the region in- and outside of the plastic hinge of the columns are summarized in Table 6. In addition, Table 8 summarizes the values adopted for each parameter required to completely define *Steel*02 model. Regarding the *Steel*02 model, the hardening modulus was considered to be 0.01, which represents the typical yielding plateau with large deformations for hot rolled steel. The value adopted for the radius of the initial nonlinear branch was lower than the recommended value R0 = 18.50 [21] for undamaged steel bars. Consequently, the energy dissipation under cyclic loading is reduced, which is consistent with the behavior of the RC elements with damaged reinforcing bars.

(3) BFRP Material Model

Through experimental observation and peeling off the cover concrete within the repair procedure, it was found that the original BFRP bars were not ruptured in the original columns. This indicates that the original BFRP bars are still working. Thus, the simulation of repaired columns should consider the original BFRP bars and NSM BFRP bars simultaneously. The mechanical properties of BFRP bars are summarized in Table 1.

### 3.3. Numerical Modeling Procedures

Based on the above introduction of the element model and the modified materials model, the numerical model can be established by the following steps. First, the geometry of the columns and the corresponding materials should be defined. Secondly, the mechanical parameters for different materials are defined according to different types of material models. Thirdly, the grid is divided for the cross section of the columns and endowing the grid with determined material parameters. Fourthly, after selecting the element model, the load is applied to the element model. Finally, according to determined calculation criteria, the results are output expectantly. In this way, the numerical model can be established for the dynamic inelastic analysis of the repaired columns under cyclic loading. More details on simulation results of the repaired columns are seen in Section 5.

### 3.4. Numerical Results

Like in tests performed with displacement control, the numerical simulation of repaired columns under the combined effects of a constant axial compression was performed applying quasi-static increasing lateral deformation reversals at the load point.

**Figure 13:**
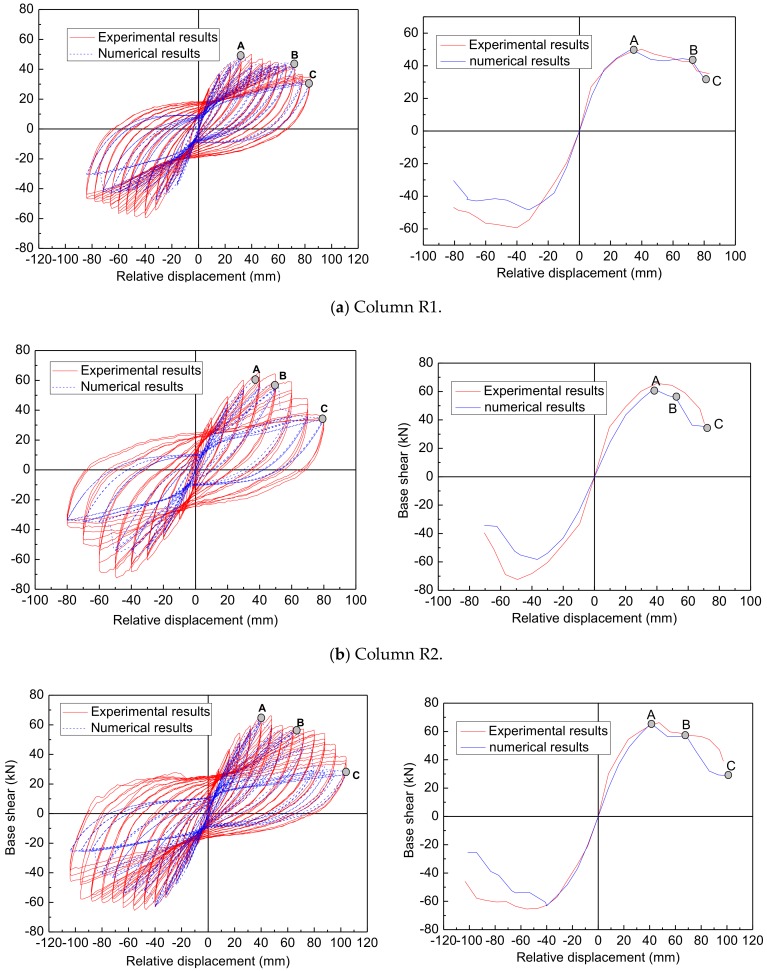

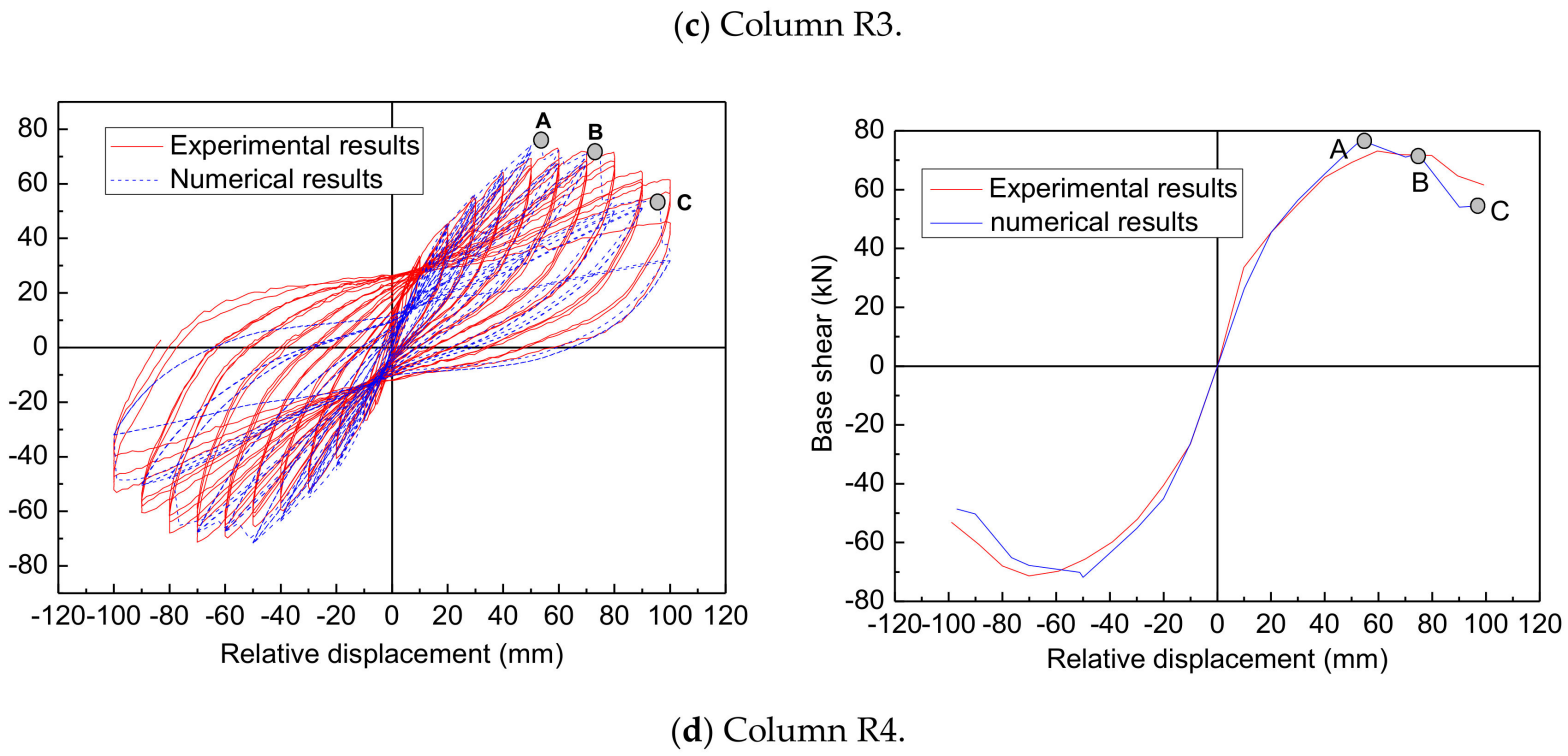
The hysteretic curves for repaired columns: (**a**) Column R1; (**b**) Column R2; (**c**) Column R3; (**d**) Column R4.

The numerical response obtained for the repaired columns and the comparison with individual tests is presented in Figure 13. It shows from this figure that the numerical and experimental hysteretic responses for the four repaired columns, and the corresponding load–displacement envelopes are also shown in Figure 10. As an example, the numerical results of column R1 are illustrated in detail.

As shown in Figure 13a, it can be observed that the fall of the loading capacity occurs twice to the load–displacement curves. The first fall of the loading capacity (point A) is resulted by the fracture of the longitudinal fibers of the BFRP sheets jacketing, and the other one (point B) is resulted by the rupture of the NSM BFRP bars. The fall points of the loading capacity for the numerical results can fit that of the experimental results. However, the fall of the loading capacity in the numerical results is sharper than that of the experimental results because in the numerical model, the FRP materials were supposed to have perfect elasticity in the loading process, but the rupture of any materials in the experiment takes place gradually, especially the FRP materials surrounded by other materials. Furthermore, as shown in Figure 13a, the column R1 behaved asymmetrically in the positive cycle (displaced to the south) and the negative cycle (displaced to the north). This can be attributed to the unsymmetrical damage in the original column and the unsymmetrical removal and replacement of loose concrete during the repair procedure. However, in the simulation, the damage and repair are assumed to be symmetrical, so the numerical results behavior symmetrically. Subsequently, the numerical results cannot fit the experimental results completely. The same phenomenon also occurs to the other three repaired columns.

It can be seen in Figure 13 that the numerical simulation accurately reproduces the global stiffness and is among the experimental responses of the repaired columns. In addition, the comparisons of the maximum lateral strength between the numerical results and the experimental results for all repaired columns are summarized in the Table 9. It is seen from Table 9 that the relative errors are all smaller than 5%, except the negative direction of columns R1 and R2. In general terms, a good agreement was found between the numerical and experimental results, in terms of maximum force as well as force and displacement evolutions. This implies the modified damage index and the corresponding proposed model are applicable to the finite element analysis of repaired columns with cumulative damage.

Legend:

A—Rupture of longitudinal fiber of BFRP sheets jacketing
B—Fracture of NSM BFRP bars
C—Ultimate displacement capacity

**Figure 14:**
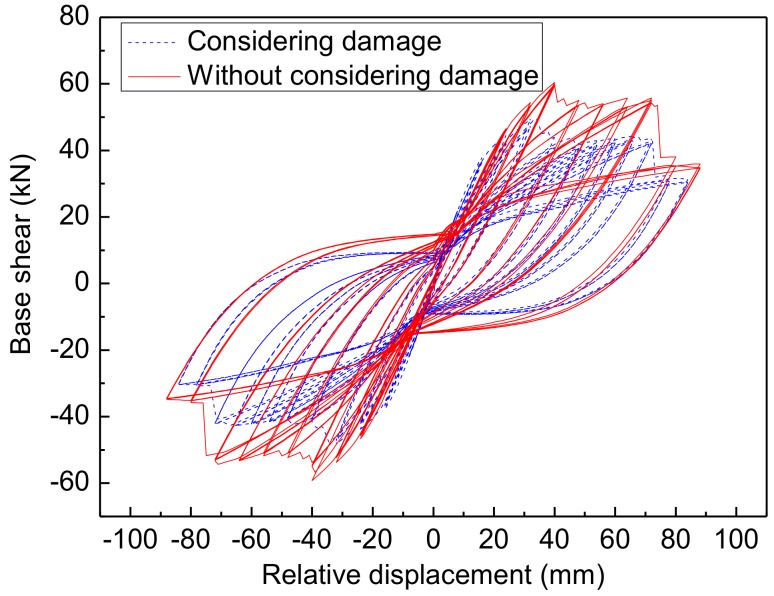
Comparison of results between considering damage and without considering damage for the repaired Column R1.

In order to illustrate the necessity of this paper, the comparison between the numerical response considering damage and numerical response without considering damage column R1 is presented in Figure 14. It shows from this figure that the two hysteretic responses for column R1 have significant differences, especially for the strength. This indicates that the original unrepaired damage must be considered in the simulation for the repaired columns after earthquake.

## 4. Evaluation of Repair Design Scheme

After the computational model is verified, the efficiency of the repair technique used in the tests should be evaluated and the deficiency of the proposed repair technique should be improved for future use. This is because the strength of the repaired columns is much higher than that of the original columns as seen in Section 3.1.

The proposed repair technique is initially designed to restore the column strength associated with the peak load in the original test. In the proposed repair technique, the NSM BFRP bars were design to restore the loss of the flexural strength due to the yielded steel bars. Furthermore, the longitudinal fibers of the bidirectional BFRP sheets helped to prevent the splitting failure of the concrete along the NSM BFRP bars, while the transversal fibers were adopted to achieve confinement to prevent the buckling failure of NSM BFRP bars. From the comparison of the maximum lateral strengths between the repaired columns and original columns summarized in Table 10, it can be found that the rise in the ratio of the NSM reinforcement while having the same fiber volumetric ratio of BFRP sheets, such as from 0.375% for column R1 to 0.586% for column R2 and from 0.375% for column R3 to 0.586% for column R4, cannot increase the lateral strength significantly, and the final increase in lateral strength are 5.43% and 1.23%, respectively. However, the maximum lateral strength of the columns increases significantly with the increase of the fiber volumetric ratio of BFRP sheets, such as from 0.675% for columns R1 and R2 to 1.125% for columns R3 and R4, the maximum lateral strength of column R3 is increased by 11.75% compared to column R1, and the maximum lateral strength of column R4 is increased by 7.55% compared to column R2. It is contributed to the longitudinal fibers of BFRP sheets, and this implies that the longitudinal fibers of BFRP sheets have a great impact on restoring the strength for the repaired columns.

**Figure 15:**
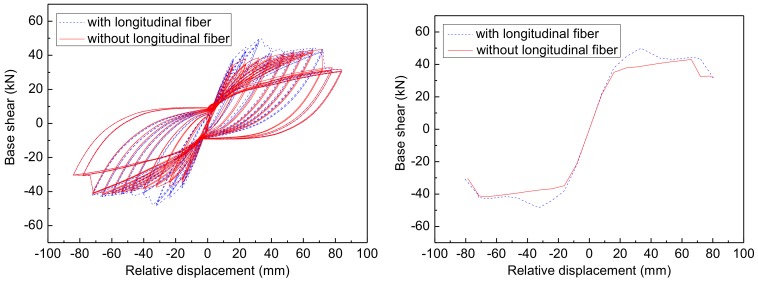
The hysteretic curves for column R1.

In order to understand the behavior of bidirectional BFRP sheets clearly, herein the column R1 is taken to analyze the effect of the longitudinal fibers of BFRP sheets on the lateral strength of the repaired columns using the proposed repair technique. The numerical responses obtained from column R1 with- or without considering longitudinal fibers are presented in Figure 15, and the corresponding maximum lateral strengths are summarized in Table 11.

From Figure 15, it is seen that the difference of numerical results between with- and without considering the longitudinal fibers gradually increase with the increase of lateral displacement when the applied lateral displacement is in the range from 20 mm to 40 mm. When the lateral displacement equals to 40 mm, the fracture of longitudinal fibers occurs to the column in the test and also occurs in the numerical results with considering longitudinal fiber. After the fracture of the longitudinal fibers, the numerical results with considering longitudinal fibers overlap that without considering longitudinal fibers.

In Table 11, it is found that the maximum lateral strength of the numerical results without considering longitudinal fibers (*V*_rn2_) is decreased by 13.66% for the positive direction and 13.76% for the negative direction compared to the maximum lateral strength of numerical results considering longitudinal fibers (*V*_rn1_). The difference between the numerical results (*V*_rn2_) and experimental results of column R1 (*V*_r_) is 14.15% for the positive direction and 29.99% for the negative direction. However, the difference between the numerical results (*V*_rn2_) without considering longitudinal fibers but only considering the effects of the NSM BFRP bars and experimental results of column O1 (*V*_o_) is 0.59% for the positive direction and −13.59% for the negative direction. This implies that the initial design can restore the lateral strength to some extent in spite of neglecting the effect of longitudinal fibers on the lateral strength. However, as a matter of fact, the longitudinal fibers of the bidirectional BFRP sheets can impact the lateral strength. Thus, the effect of the longitudinal fibers of BFRP sheets needs to be taken into account in the simulation of the damaged columns repaired by using the proposed repair technique.

The comparisons between the experiment results of the original columns and the numerical results of the repaired columns without considering the longitudinal fibers are also made for the other three columns, and the corresponding maximum lateral strengths for the four columns are all shown in Table 12. It is found that except the negative direction of columns R1 and R2, the strength of the repaired columns was restored or even enhanced compared to that of the original columns. This implies that the initial repair design only considering the effect of the NSM BFRP bars on the flexural strength can restore the lateral strength associated with the peak load. Subsequently, the NSM BFRP bars technique is effective in restoring the flexural strength if the buckling failure of NSM reinforcement can be avoided; therefore, the bidirectional BFRP sheets are unnecessary, and unidirectional BFRP sheets may be another good choice because the unidirectional BFRP sheets have higher strength, stiffness, and ultimate strains than bidirectional BFRP sheets but have the lower cost [41]. In this way, the amount of unidirectional BFRP sheets is lower than the bidirectional BFRP sheets by using the equal strength to replace each other. Thus, only to restore the peak strength, the combination of the NSM BFRP bars and external unidirectional BFRP sheets is higher than the combination of the NSM BFRP bars and external bidirectional BFRP sheets in the cost-efficiency.

## 5. Conclusions

This paper develops a computational model for the numerical analysis of repaired bridge columns in which a repair technique based on combination of NSM BFRP bars with external BFRP sheets jacketing is used to restore the flexural strength. The computational model takes into account the existing original damage of the repaired columns by using a modified material damage-accumulation model proposed to modify the constitutive of concrete and steel bar. The damage-accumulation models take into account the strength and stiffness reduction with the increase of external loading and the appearance of softening behavior. Then the computational model is implemented into the OpenSees finite element program and verified by experimental data. Furthermore, based on the proposed computational model, the efficiency of the repair technique used in tests is evaluated. Some conclusions can be drawn as follows:

(1) The proposed damage-accumulation model is simple and effective in evaluating the residual strength and stiffness of the materials of bridge columns under cyclic loading.
(2) The developed computational model is verified by comparing the numerical results with experimental data. A good agreement between the numerical and experimental results in terms of the load–displacement curves (initial stiffness, maximum strength) shows that the behavior of the repaired columns can well represent with the numerical model.
(3) Based on the evaluation results of efficiency for the repair technique, it is found that the proposed design method is effective in calculating the amount of NSM BFRP bars for the repaired columns. Furthermore, the NSM BFRP bars can effectively restore the flexural strength of the repaired columns. Thus, only to restore the peak strength, the combination of the NSM BFRP bars and external unidirectional BFRP sheets is higher than the combination of the NSM BFRP bars and external bidirectional BFRP sheets in the cost-efficiency.

Experimental data is essential for the development and calibration of reliable numerical models for the assessment of the seismic capacity of existing RC structures. However, data on the response of repaired columns damaged previously are still scarce. Additional experimental work needs to be investigated on covering all aspects and variables that influence the cyclic behavior of this type of elements and structures.

## Figures and Tables

**Table 1:** Mechanical properties of materials.

Material	Elastic Modulus (GPa)	Yield Stress (MPa)	Tensile Strength (MPa)	Ultimate Strain (%)
Steel bar	200	390	510	14.5
BFRP bar	50	-	1095	2.19
BFRP sheet	39.5 (42.9)	-	550 (528)	1.28 (1.34)

Note: for the basalt fiber reinforced polymer (BFRP) sheet, the parenthetical figure following 528, 42.9, 1.34 is of the longitudinal BFRP sheet.

**Table 2:** The repairing program using the BFRP bars-sheets composite repair technique.

No. of Original Column	Treatment Method of Surface	Type after Treatment	No. of Repaired Column	NSM-BFRP Bar		BFRP Sheet	
				Diameter (mm)	Ratio of NSM Reinforcement *ρ_f_* (%)	Number of Layers	Fiber Volumetric Ratio *ρ_fv_* (%)
O1	-	RC column	R1	8	0.375	3	0.675
O2	Peeling off BFRP sheets	RC column	R2	10	0.586	3	0.675
O3	-	Hybrid reinforced column	R3	8	0.375	5	1.125
O4	Peeling off BFRP sheets	Hybrid reinforced column	R4	10	0.586	5	1.125

**Table 3:** Comparison of the results for the original and corresponding repaired columns.

Column Notation	Lateral Strength (kN)		Service Stiffness (kN/mm)		Ductility Capacity (mm/mm)	
	Original *V*_o_	Repaired *V*_r_	Original *K*_o_	Repaired *K*_r_	Original *D*_o_	Repaired *D*_r_
Column 1	45.43	54.74	2.55	2.53	4.63	4.29
Column 2	54.31	68.39	3.46	3.53	5.53	4.27
Column 3	49.58	65.56	2.38	2.63	4.04	4.53
Column 4	53.64	71.60	2.86	2.48	5.59	3.82

**Table 4:** Response indices for the repaired columns.

Column Notation	Strength Index 100 × *V*_r_*/V*_o_ (%)	Stiffness Index 100 × *K*_r_*/K*_o_ (%)	Ductility Index 100 × *D*_r_/*D*_o_ (%)
Column 1	120.49	98.93	92.83
Column 2	125.92	102.22	77.32
Column 3	132.24	110.14	112.04
Column 4	133.47	86.86	68.44

**Table 5:** The ultimate rotation and drift for all repaired columns.

Column	O1	O2	O3	O4
*θ*_u_ (1/rad)	0.0563	0.0548	0.0778	0.0757
*d*^’^_u_ (mm)	85	83	117	114

**Table 6:** Mechanical properties of concrete and steel bars considering damage.

Column	*D*’_c_	*D*’_s_	*f*_c_’ (MPa)	*E*_c_’ (MPa)	*f*_y_’ (MPa)	*E*_s_’ (MPa)
O1	0.834	0.975	12.925	14594.064	287.280	170735.172
O2	0.320	0.854	15.240	15880.162	300.069	174378.637
O3	0.895	0.985	12.647	14439.611	286.273	170448.112
O4	0.675	0.947	13.640	14991.219	290.280	171589.866

**Table 7:** Concrete mechanical properties adopted in the numerical model (unconfined and confined).

Concrete	Confine Condition	*E* (MPa)	*f*_cm_ (MPa)	*ε*_0_ (‰)	*f*_cmu_ (MPa)	*ε*_u_ (‰)
Original concrete	Unconfined	16,680	16.68	2.0	3.336	6.0
	Confined	21,050	21.05	2.52	6.315	30.0
Repaired concrete	Unconfined	23,056	23.056	2.0	4.611	6.0
	Confined	29,097	29.097	2.52	8.729	30.0

**Table 8:** *Steel*02 model parameters considered in the column numerical modeling.

Material Model	Parameter	Value
*Steel*02	Hardening modulus	0.01
	R0	14.0
	cR1	0.925
	cR2	0.15

**Table 9:** Comparison of the strength between the experiment and numerical analysis.

Column	Column R1		Column R2		Column R3		Column R4	
	Positive	Negative	Positive	Negative	Positive	Negative	Positive	Negative
Experiment F_e_ (kN)	50.05	−59.42	65.68	−72.41	66.22	−65.42	73.13	−71.32
Numerical F_n_ (kN)	49.77	−48.24	61.71	−58.26	65.54	−63.21	76.77	−71.80
Relative error 100*(F_e_−F_n_)/F_e_ (%)	0.56	18.81	6.05	19.54	1.03	3.37	4.97	0.68

**Table 10:** Comparison of results.

Column Notation	Lateral Strength (kN)		100 × *V*_r_/*V*_o_ (%)
	Original (*V*_o_)	Repaired (*V*_r_)	
Column O1/R1	45.43	54.74	120.49
Column O2/R2	54.31	68.39	125.92
Column O3/R3	49.58	65.56	132.24
Column O4/R4	53.64	71.60	133.47

**Table 11:** Comparison of experimental and numerical maximum lateral strength for column R1.

Loading Direction	Experiment (kN)		Numerical (kN)		100 × (*V*_rn1_ − *V*_rn2_)/*V*_rn1_(%)	100 × (*V*_r_ − *V*_rn2_)/*V*_r_(%)	100 × (*V*_o_ − *V*_rn2_)/*V*_o_(%)
	Original *V*_o_	Repaired *V*_r_	With Longitudinal Fiber *V*_rn1_	Without Longitudinal Fiber *V*_rn2_			
Positive	42.72	50.05	49.77	42.97	−13.66	−14.15	0.59
Negative	−48.14	−59.42	−48.24	−41.60	−13.76	−29.99	−13.59

**Table 12:** Comparison of experimental and numerical maximum lateral strengths for four repaired columns.

Column	Direction	Experiment *V*_o_ (kN)	Numerical *V*_rn2_ (kN)	100 × (*V*_rn2_ − *V*_o_)/*V*_o_ (%)
R1	Positive	42.72	42.97	0.59
	Negative	−48.14	−41.6	−13.59
R2	Positive	50.15	54.63	8.93
	Negative	−58.48	−53.27	−8.92
R3	Positive	47.92	55.04	14.84
	Negative	−51.27	−52.64	2.67
R4	Positive	49.30	67.05	36.00
	Negative	−57.99	−64.09	10.54
